# Fully Automated ^68^Ga-Labeling and Purification of Macroaggregated Albumin Particles for Lung Perfusion PET Imaging

**DOI:** 10.3389/fnume.2021.778191

**Published:** 2021-11-18

**Authors:** Frédérique Blanc-Béguin, Julien Masset, Philippe Robin, Raphaël Tripier, Simon Hennebicq, Valérie Guilloux, Charles Vriamont, Corentin Warnier, Virginie Cogulet, Peter Eu, Pierre-Yves Salaün, Pierre-Yves Le Roux

**Affiliations:** ^1^Medecine Nucleaire, CHRU Brest, EA3878 (GETBO) IFR 148, Univ Brest, Brest, France; ^2^Trasis Radiopharmacy Instruments, Ans, Belgium; ^3^Univ Brest UMR-CNRS 6521 (CEMCA), IFR 148, Brest, France; ^4^Medecine Nucleaire, CHRU Brest, Brest, France; ^5^Univ Brest, EA3878 (GETBO), INSERM 1078, Brest, France; ^6^Pharmacie, CHRU Brest, INSERM 1078, Brest, France; ^7^Cancer Imaging, Peter MacCallum Cancer Centre, Melbourne, VIC, Australia

**Keywords:** [^68^Ga]Ga-MAA, gallium-68, automated synthesis, automated purification, perfusion PET/CT

## Abstract

Lung PET/CT is a promising imaging modality for regional lung function assessment. Our aim was to develop and validate a fast, simple, and fully automated GMP compliant [^68^Ga]Ga-MAA labeling procedure, using a commercially available [^99m^Tc]Tc-MAA kit, a direct gallium-68 eluate and including a purification of the [^68^Ga]Ga-MAA.

**Method:** The synthesis parameters (pH, heating temperature) were manually determined. Automated ^68^Ga-labeling of MAA was then developed on a miniAIO (Trasis®, Ans, Belgium) module. An innovative automated process was developed for the purification. The process was then optimized and adapted to automate both the [^68^Ga]Ga-MAA synthesis and the isolation of gallium-68 eluate required for the pulmonary ventilation PET/CT.

**Results:** The 15-min process demonstrated high reliability and reproducibility, with high synthesis yield (>95 %). Mean [^68^Ga]Ga-MAA radiochemical purity was 99 % ± 0.6 %. The ^68^Ga-labeled MAA particles size and morphology remained unchanged.

**Conclusion:** A fast, user friendly, and fully automated process to produce GMP [^68^Ga]Ga-MAA for clinical use was developed. This automated process combining the advantages of using a non-modified MAA commercial kit, a gallium-68 eluate without pre-purification and an efficient final purification of the [^68^Ga]Ga-MAA may facilitate the implementation of lung PET/CT imaging in nuclear medicine departments.

## Introduction

Ventilation-perfusion (V/Q) PET/CT (Positron Emission Tomography/Computed Tomography) is a promising imaging modality for regional lung function assessment. The same carrier molecules as conventional V/Q scan are used (i.e., carbon nanoparticles for ventilation and macro aggregated albumin (MAA) for perfusion), but they are labeled with gallium-68 instead of technetium-99m (1). Thereby, V/Q PET/CT and V/Q SPECT/CT imaging assesses similar physiological processes, but with the technical advantages of PET over conventional SPECT imaging, including higher sensitivity, spatial and temporal resolution, and speed of acquisition (2, 3). The transition from SPECT to PET technology showed promising results in a variety of pulmonary diseases, including pulmonary embolism diagnosis (4, 5), assessment of pulmonary reserve in lung cancer patients before surgery (6), radiotherapy planning to maximize dose to the tumor while minimizing the dose to the surrounding lungs (7–10), or surgical evaluation of patients undergoing lung volume reduction surgery (11). Other advantages of PET imaging include the reduction of the acquisition time (~2–3 min) and a greater access to respiratory gated acquisition (1). Furthermore, the use of gallium-68 could be a suitable alternative to prevent production delays due to ^99^Mo/^99m^Tc generator shortage (12).

While ^68^Ga-labeled carbon nanoparticles production can be readily performed using an unmodified commercially available Technegas generator (Cyclopharm Ltd., Australia) (13), the radiolabeling of the MAA is logistically less straightforward with gallium-68 as compared with technetium-99m. Indeed, the ^99^Mo/^99m^Tc generator is eluted with 0.9% NaCl whereas the ^68^Ge/^68^Ga generator is eluted with HCl which is not suitable for intravenous perfusion. Moreover, the gallium-68 eluate contains free metal impurities and germanium-68 breakthrough. Therefore, the [^68^Ga]Ga-MAA needs to be purified prior to injection. Several radiolabeling processes have been proposed, with various options in the key stages of the process (the source of MAA, pre-purification of the gallium-68 eluate, purification of the final product). First, most groups used MAA included in commercial MAA kits for technetium-99m, which has the advantage of simplicity and availability (14–16). Other groups performed a centrifugation stage to eliminate free albumin from the kits (5, 12, 17–20). Second, some groups performed a pre-purification of the gallium-68 eluate to improve the radiolabeling yield (5, 15, 16). Third, a critical stage is the purification of the [^68^Ga]Ga-MAA at the end of the synthesis to separate [^68^Ga]Ga-MAA from any other chemicals, including free gallium-68. In that respect, the use of sep-Pak C18 cartridge (17) or centrifugation (12, 18, 20) was tested. The main drawbacks of these processes are that they are time consuming, increase the radiation dose for the operators, and increase the risk of bacterial contamination. Moreover, the radiolabeling yields are reduced with the use of sep-Pak C18 cartridge (17) and the centrifugation is not prone to automation. Finally, most groups proposed a manual process of MAA labeling with gallium-68 (5, 12, 16–20). The automation of the labeling procedures is of high interest to minimize the operator's radiation exposure, decrease the procedure time, and limit the human errors.

For these considerations, and to expedite the implementation of lung perfusion PET/CT imaging in nuclear medicine departments, the development of a simple, rapid, and fully automated process for ^68^Ga-labeling of macroaggregated albumin particles would be of great interest. Automation with the use of disposable cassettes should maximize the pharmaceutical product quality, minimize potential cross-contaminations, reduce operator radiation dose, and avoid time-consuming cleaning procedures. To ensure patient's safety, the labeling procedure should be compliant with Good Manufacturing Practices (GMP). Moreover, a few milliliters of gallium-68 eluate could be isolated during the MAA labeling process for the ^68^Ga-labeling of carbon nanoparticles for ventilation PET imaging. Finally, the process should demonstrate reliability and reproducibility.

Our aim was to develop and validate a user friendly and fully automated GMP compliant [^68^Ga]Ga-MAA labeling procedure, from an unmodified commercially available [^99m^Tc]Tc-MAA kit and a direct gallium-68 eluate, including an efficient final purification of the [^68^Ga]Ga-MAA.

## Materials and Methods

### General

For the labeling procedure, the reagents and solvents were of the highest grade and were used without further purification. Ultrapur 0.1N HCl for generator elution was purchased from Eckert & Ziegler Radiopharma Gmbh (Berlin, Germany). The sodium acetate solutions were prepared from EMSURE® ACS anhydrous sodium acetate and EMSURE® water for analysis, both purchased from Merck KGaA (Darmstadt, Germany).

The sodium acetate solutions were prepared and sterilized on a 0.22-μm pore size filter, in a class II biological safety cabinet. Macoflex® isotonic 0.9% NaCl was purchased from MacoPharma (Tourcoing, France). The commercial [^99m^Tc]Tc-MAA kits (Pulmocis®) were purchased from CIS Bio International (CURIUM, Saclay, France). The commercial kits contained a sterile, nonpyrogenic, lyophilized mixture of 2.0 mg of macroaggregated albumin, human albumin, stannous chloride (SnCl_2_. 2H_2_O), and sodium chloride. The gallium-68 was obtained as [^68^Ga]Ga-chloride from a 1.85 GBq commercially available TiO_2_ based ^68^Ge/^68^Ga generator (GalliaPharm®), produced by Eckert & Ziegler Radiopharma Gmbh (Berlin, Germany) under GMP and distributed by CycloPharma (CURIUM, S^t^ Beauzire, France).

For the radiochemical purity control, a 0.1 M sodium citrate solution was prepared from distilled water and sodium citrate anhydrous purchased from Honeywell Fluka™ and distributed by Fisher Scientific (Illkirch, France).

### Manual Procedure: Determination of Synthesis Parameters

Based on previously published data (5, 12, 16–18), four pH values (3.8, 4.3, 4.8, 5.3) and four heating temperatures (50, 60, 70, and 80°C) were tested. The commercial MAA kits were manually suspended either in 2.0 mL of 0.31 M, 0.35 M, 0.39 M, or 0.43 M sodium acetate solution to obtain a reaction mixture with pH of 3.8, 4.3, 4.8, and 5.3, respectively. Then 5 mL of 0.1 N HCl were introduced in the vials. The reaction mixture pH was measured with a pH meter (HANNA Instruments, Lingo Tanneries, France). Each reaction vial was heated in a heat block for 15 min at each temperature mentioned above. At the end of the heating stage, the organoleptic properties of each suspension were visually analyzed.

### Automated Procedure Description

#### Synthesis Parameters Validation With Automated Procedure and Purification Stage Optimization

To perform the automated process, a disposable cassette was used on a miniAIO® (Trasis, Ans, Belgium) placed in a hot cell equipped with vertical laminar flow to ensure the preparation's safety according to the GMP. The software Trasis Supervision® (Trasis, Ans, Belgium) was used to program the automated reagent's transfers to label the MAA.

**Figure 1:**
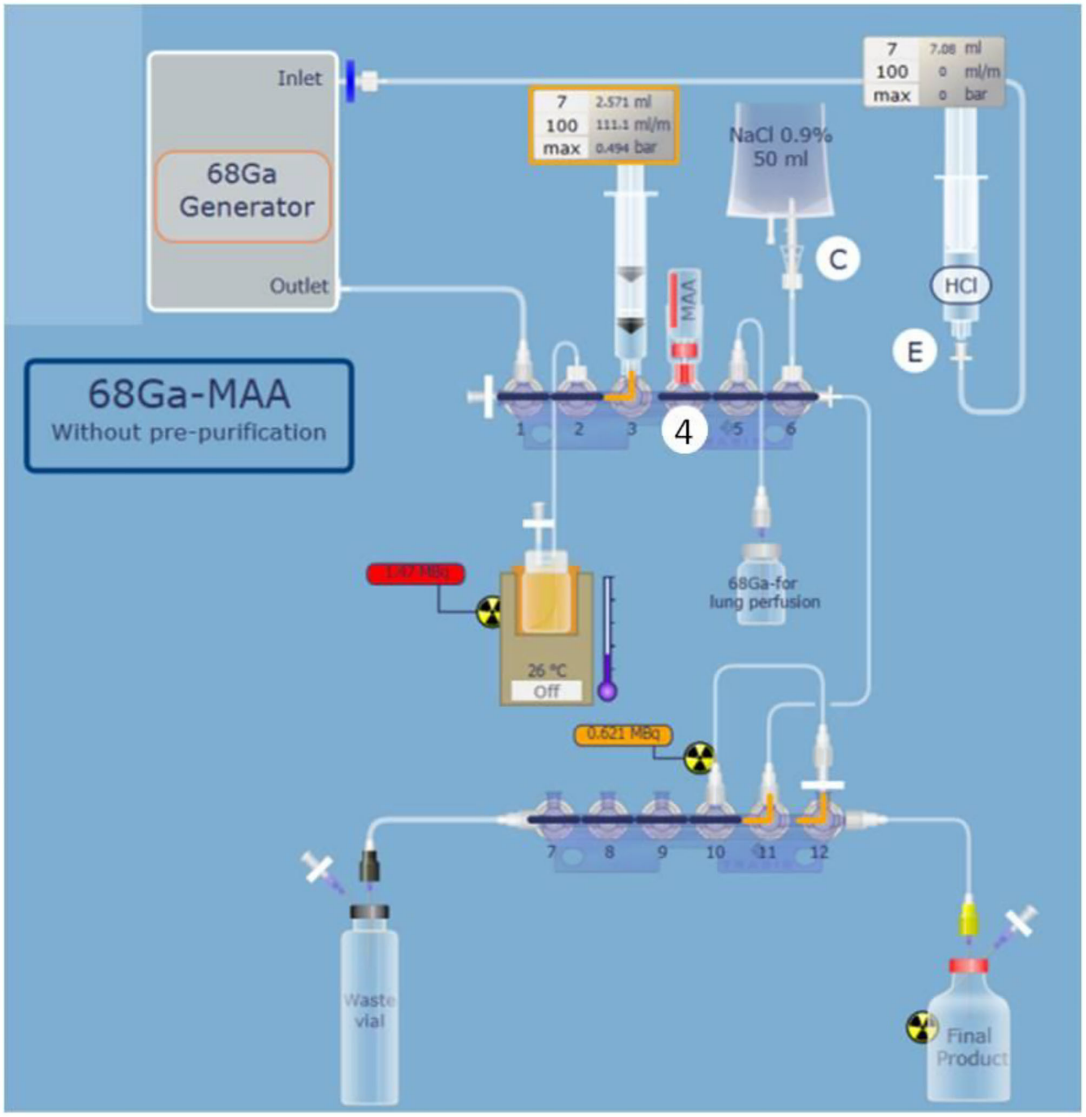
Schematic representation of complete and optimized automated 68Ga-labeling of MAA on mini AIO module with positions of reagent vials. MAA suspended in sodium acetate solution are placed in position 4 (D on the schema), 0.9% NaCl is placed in position 6. The [^68^Ga]Ga-MAA purification unit is placed in position 12.

The commercial MAA kit was manually suspended in 2.0 mL of sodium acetate solution. Then the vial was placed on the cassette position number 4 (Figure 1). The first stage of the automated procedure was the transfer of the suspended MAA from the commercial vial to the reactor. Then, the ^68^Ge/^68^Ga generator was eluted with 5 mL of 0.1 N HCl and the eluate was added to the reactor. The generator was eluted 24 h prior to the labeling procedure to eliminate zinc-68, which can affect the yield. The final volume of the reaction mixture was approximately 7 mL. The reactor was heated for 15 min. At the end of the heating stage, the product was diluted to 9 mL with 0.9% NaCl. To validate the synthesis parameters manually determined, radiolabeling yields and radiochemical purity controls were performed on three syntheses without purification stage. Indeed, the labeling parameters and the purification stage both influence the synthesis yield. So, in order to evaluate the purification stage efficiency by using the synthesis yield, the labeling parameters had to be validated first without purification.

**Figure 2:**
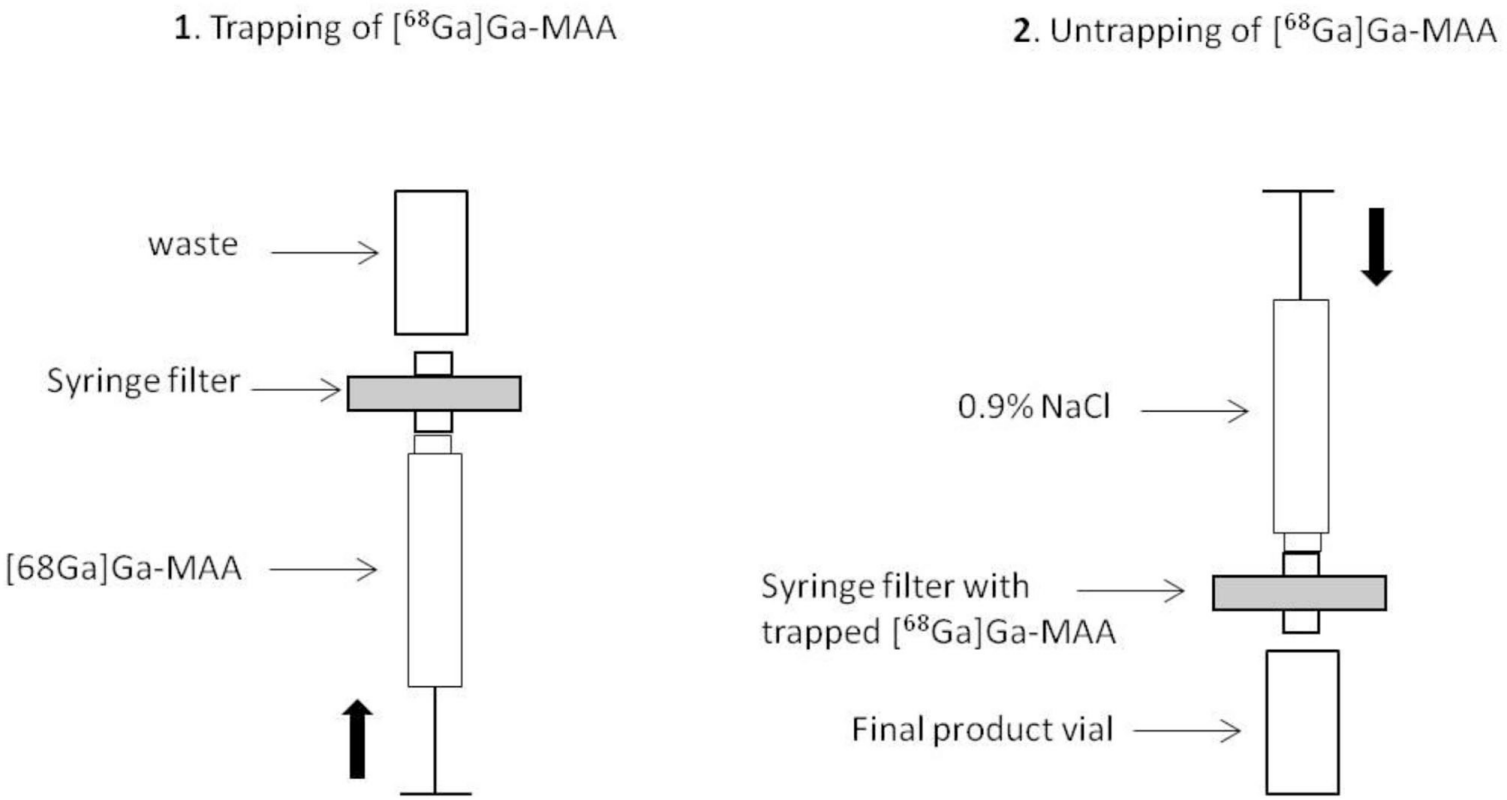
Schematic representation of the [^68^Ga]Ga-MAA purification stage.

A novel procedure was tested for the purification step. The final suspension was passed through a low protein binding filter to purify the obtained [^68^Ga]Ga-MAA. Various types of filters were tested to optimize the purification stage: a 5-μm pore size filter, a 0.45-μm pore size filter, a 0.22-μm pore size double membrane filter, and a 0.22-μm pore size ventilated filter. In order to improve the release of [^68^Ga]Ga-MAA from the filter, the suspension was passed through the filter from the bottom to the top with as little air as possible. Then, the [^68^Ga]Ga-MAA was removed from the filter and transferred into the final vial by passing 10 mL of 0.9% NaCl from the top to the bottom of the filter (Figure 2). For each filter tested, the release yield of [^68^Ga]Ga-MAA from the filter was assessed as follows: radioactivity of the filter/(final [^68^Ga]Ga-MAA suspension radioactivity + radioactivity of the filter).

#### Automated Procedure Optimization for Clinical Use

Once the synthesis parameters were determined, the automated synthesis process was adjusted to isolate 2 mL of gallium-68 for carbon nanoparticles labeling (ventilation PET imaging) and to use the remaining 3 mL of gallium-68 eluate for the MAA labeling. For this purpose, the molarity of the sodium acetate solution was adjusted to respect the reaction media pH validated before. The commercial MAA kit was manually suspended in 2.0 mL of the sodium acetate solution and connected on the cassette position number 4. Then, the automated process was performed as follows: first, the MAA suspension was transferred into the reaction vial. Then, the ^68^Ge/^68^Ga generator was eluted with 5 mL of 0.1 M HCl while the eluate was collected in the syringe placed on the cassette position number 3 (Figure 1). When the 5 mL of eluate were contained in the syringe, 2 mL were transferred in an independent vial for carbon nanoparticles labeling and 3 mL of gallium-68 eluate were introduced in the reaction vial which already contained the MAA suspension. The reaction vial was heated at 60°C to label the MAA. To improve the labeling yield by saving time and reducing the loss of activity due to radioactivity decay, three heating times were tested (15, 10, and 5 min). Labeling times tested were selected given the previously published data (5, 12, 16–18). The choice of the reactor heating time was based on syntheses yield, [^68^Ga]Ga-MAA suspension's radiochemical purity, and the percentage of particles inferior to 3 μm results obtained with the three times tested.

### Validation of the Process for Clinical Use

The validation of the labeling procedure for clinical use was performed by evaluating the reliability and reproducibility of the process, followed by quality controls on the obtained [^68^Ga]Ga-MAA suspensions.

To assess the reliability and reproducibility of the process, an automated procedure for clinical use was performed twice in triplicate. The ^68^Ge/^68^Ga generator and the sodium acetate solution were changed between the two triplicates. Calculation of yields was based on the amount of radioactivity in the gallium-68 eluate at the start of the process and in the product obtained at the end of the process (with or without [^68^Ga]Ga-MAA purification stage). At the end of the process, the purification step efficiency was assessed by determining the percentage of radioactivity remaining on the purification filter.

[^68^Ga]Ga-MAA suspensions were tested according to standards applied for clinical use [i.e., EU Pharmacopeia monograph for [^68^Ga]Ga-radiopharmaceuticals and [^99m^Tc]Tc-macroagregated albumin] to validate the final process for clinical production (*n* = 6).

**Figure 3:**
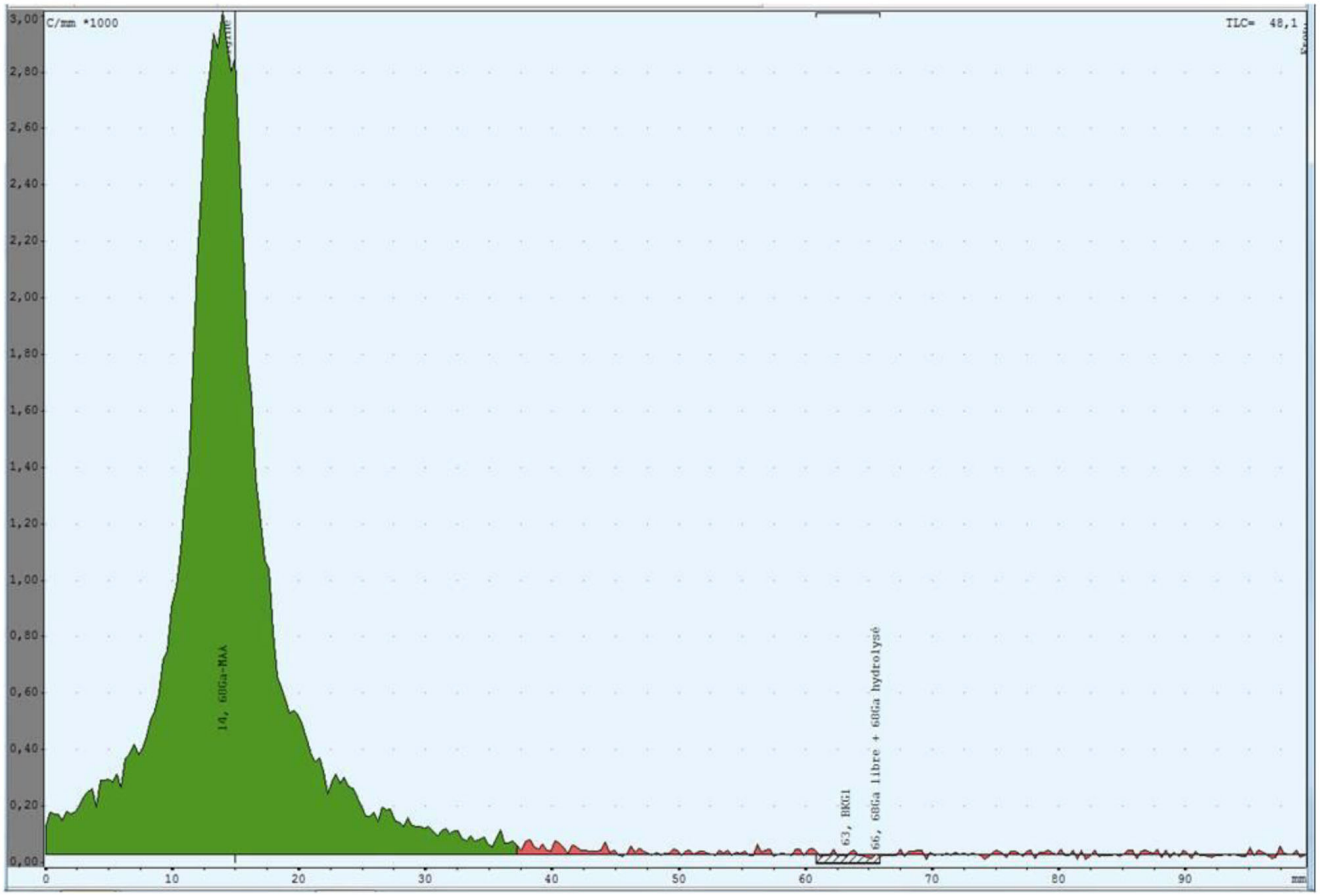
Thin layer chromatography of [^68^Ga]Ga-MAA suspension produced with the process for clinical use. The green region is the radioactivity due to the [^68^Ga]Ga-MAA.

The radiochemical purity of [^68^Ga]Ga-MAA suspension was assayed using instant thin-layer chromatography (iTLC) on ITLC-SG glass fiber sheets from PALL Life Sciences (Port Washington, NY) recorded with Gina Star TLC and analyzed using miniGita software from Raytest (Straubenhardt, Germany) and distributed by Elysia (Angleur, Belgium). The percentages of each fraction were determined relative to the total activity of the chromatogram (Figure 3). A 0.1 M sodium citrate solution at pH 5 was the mobile phase.

The radioactivity distribution was determined by filtration of the [^68^Ga]Ga-MAA suspension through a 3-μm pore size Whatman® Nuclepore™ track-Etched polycarbonate membrane (Merck GaA, Darmstadt, Germany). The filter and filtrate radioactivity were measured using a 3″ × 3″ NaI(Tl) pinhole gamma counter (Canberra, Montigny-Le-Bretonneux, France).

The radionuclidic purity determination was performed after gallium-68 decay (at least 48 h) using a 3″ × 3″ NaI(Tl) pinhole gamma counter (Canberra, Montigny-Le-Bretonneux, France) in order to quantify germanium-68 percentage.

The pH of the [^68^Ga]Ga-MAA suspension was controlled using pH test paper. The sterility and endotoxins level of the product were investigated by the hygiene department of Brest University Hospital after gallium-68 decay. According to the monograph 20,601 of pharmacopeia (9th edition), the sterility of the final product was tested by inoculing 9.5 mL of trypticase soy broth and 9.5 mL of thioglycolate broth with 0.5 mL of [^68^Ga]Ga-MAA suspension each. Both broths were incubated 14 days at 22 and 30°C, respectively.

The endotoxins level was assessed using the Endosafe®-Nexgen PTS (Charles River, Ecully, France) colorimetric technique.

[^68^Ga]Ga-MAA *in vitro* stability was controlled by the determination of the radiochemical purity and the radioactive distribution 3 h (= T3h) after the end of the labeling process and were compared with the values at T0.

### Labeled MAA Integrity at the End of the Labeling

To investigate the MAA integrity at the end of the labeling procedure, the size and morphology of labeled-MAA were studied.

The size was measured using an optical microscope Leica DM 1,000 LED (Leica Microsystemes SAS, Nanterre, France) and a hemacytometer (Ozyme, St Quentin-en-Yvelines, France). Three samples were investigated and compared with the applied standards for clinical use (i.e., EU Pharmacopeia monograph for [^99m^Tc]Tc-macroagregated albumin).

The morphology was investigated on MAA labeled with natural gallium-69 (0.018 nmol of ^69^Ga in 5 mL of 0.1 N HCl) instead of gallium-68. The morphology of [^69^Ga]Ga MAA was examined using a HITACHI S-3200N SEM (HITACHI, Tokyo, Japan) and was compared with the MAA morphology of the commercial kit suspended in 0.9% NaCl.

### Labeled MAA Suspension Tin Quantity

The tin dosage was performed on four [^68^Ga]Ga-MAA samples produced with the automated process for clinical use. The objective was to evaluate the purification step impact on removing tin from the final [^68^Ga]Ga-MAA suspension. The [^68^Ga]Ga-MAA samples were transferred in cleaned metal-free Teflon beakers. The weight of each sample was measured. The samples were acidified with 2 mL of 14 N ultrapure nitric acid and heated at 100°C for 24 h to proceed with the mineralization. Then samples were evaporated to dryness and suspended in 5 mL of 6 N HCl to constitute stock solutions.

Tin concentrations were measured using high resolution inductively coupled plasma mass spectrometry (HR-ICP-MS, Element XR, Thermofisher Scientific) located at Pôle de Spectrométrie Océan (PSO, Brest, IUEM, France). Five milliliters of the stock solutions were weighted and diluted with 10 mL of 2.5% nitric acid containing an internal standard (Indium at 1 ppb). Concentrations of the samples were calibrated using external calibration standards and a procedural blank was analyzed with the samples and was below the detection limit (<0.002 ppb).

## Results

### Manual Procedure: Determination of Synthesis Parameters

Organoleptic properties of MAA suspensions according to the heating temperature and the reaction media pH are shown in Table 1. At a pH of 3.8, the reaction mixture could be heated 15 min from 50 to 80°C while conserving a white suspension without aggregation. At pH of 4.3 and 4.8, the organoleptic properties of the reaction mixture were maintained at all temperatures but 80°C. With a pH of 5.3, suspended filaments were observed in the suspension at 60°C and above. Based on these results, the selected parameters were the following: a pH set at 4.3 for the reaction mixture and a labeling temperature of 60°C.

**Table 1:** Organoleptic properties of MAA suspensions according to heating temperature and reaction media pH.

**Heating temperature (^**°**^C)**	**Suspension organoleptic properties**			
	**pH = 3.8**	**pH = 4.3**	**pH = 4.8**	**pH = 5.3**
80	White suspension without sedimentation	Fast sedimentation	Fast sedimentation	Fast sedimentation
70	White suspension without sedimentation	White suspension without sedimentation	White suspension without sedimentation	Suspended filaments
60	White suspension without sedimentation	White suspension without sedimentation	White suspension without sedimentation	Suspended filaments
50	White suspension without sedimentation	White suspension without sedimentation	White suspension without sedimentation	White suspension without sedimentation

*The commercial MAA kits were suspended in 5 mL of 0.1 N HCl and 2 mL of sodium acetate solution of various molarity (0.31 M for pH = 3.8, 0.35 M for pH = 4.3, 0.39 M for pH = 4.8, 0.43 M for pH = 5.3). All suspensions were heated for 15 min*.

### Automated Procedure Description

#### Synthesis Parameters Validation With Automated Procedure and Purification Stage Optimization

Mean decay-corrected [^68^Ga]Ga-MAA radiolabeling yields obtained with the automated procedure without purification stage were 87.3 ± 2.08% (see Supplementary Table 1). Mean [^68^Ga]Ga-MAA radiochemical purity was 97.8% ± 2.0% (see Table 2).

**Table 2:** Quality control results for three [^68^Ga]Ga-MAA suspensions obtained with the process using 5 mL of gallium-68 eluate and two series of three [^68^Ga]Ga-MAA suspensions obtained with the process using 3 mL of gallium-68 eluate.

	**Synthesis number**	**Radiochemical purity (%) at T0**	**Particles <3 μm (%) at T0**	**Radionuclidic purity (%)**	**pH**	**Sterility**	**Endotoxin's level (IU/mL)**	**EOS (min)**	**Initial activity (MBq)**	**Radioactivity of final product at EOS (MBq)**
Synthesis process with 5 mL of [^68^Ga]GaCl_3_	1	95.5								
	2	99.1								
	3	98.7								
	Mean	97.8								
	Standard deviation	2.0								
Synthesis process with 3 mL of [^68^Ga]GaCl_3_	1a	100.0	0.9	>99.999	4.4	Sterile	<5	15	692	480
	2a	99.3	1.3	>99.999	4.4	Sterile	<5	15	690	511
	3a	98.4	1.1	>99.999	4.4	Sterile	<5	15	673	503
	1b	98.6	2.2	>99.999	4.4	Sterile	<5	15	895	670
	2b	99.0	0.8	>99.999	4.4	Sterile	<5	15	900	679
	3b	98.8	1.3	>99.999	4.4	Sterile	<5	15	664	488
	Mean	99.0	1.3							
	Standard deviation	0.6	0.5							

*Radiochemical purity controls of the products obtained with the first process were performed before the purification stage. The ^68^Ge/^68^Ga generator and the sodium acetate were changed between the two series (a and b) of three syntheses each (1, 2, 3) produced with the process for clinical use. EOS, end of the synthesis*.

Various filters for the purification stage were then tested. Table 3 shows the synthesis yield and MAA release percentage at the end of the synthesis with those filters. Out of the four types of filters tested, the 0.22-μm pore size ventilated filter provided the best MAA release percentage (99.5%) and the best decay-corrected synthesis yield (71%). The 0.22-μm pore size ventilated filter was therefore chosen for the purification stage.

**Table 3:** Radiolabeling yield and MAA release percentage at the end of the synthesis depending on the filter type used for the purification stage.

	**Non-vented**	**Non-vented**	**Double membrane**	**Vented**
Pore size	5 μm	0.45 μm	0.22 μm	0.22 μm
Filter diameter	25 mm	13 mm	22 mm	22 mm
Radiolabeling yield (%)	52	20	59	71
MAA release (%)	62.4	23	65.5	99.5

#### Automated Procedure Optimization for Clinical Use

The automated procedure was then optimized and adapted for clinical use. Results of syntheses performed with three radiolabeling times (5, 10, and 15 min) are shown in Table 4. The three durations did not show any significant radiolabeling yield variation. The heating stage was therefore set to 5 min to increase the non-decay-corrected yield. In order to isolate 2 mL of gallium-68 eluate for the ventilation PET study, the process was modified to only use 3 mL of gallium-68 eluate for MAA labeling. To be aligned with the best conditions determined during the manual optimization, 2 mL of 0.2 M sodium acetate solution were used to reconstitute the MAA commercial kit to reach a pH of 4.3. Following this procedure, the automated process of MAA labeling with gallium-68 took 15 min from the beginning of the generator elution to the end of the [^68^Ga]Ga-MAA suspension transfer in the final vial.

**Table 4:** Synthesis yields, results of radiochemical purity and percentage of particles inferior to 3 μm obtained with the three tested radiolabeling times (= heating time).

**Radiolabeling time in min**	**Synthesis yield in %**	**Radiochemical purity (%)**	**Particles <3 μm (%)**
5	93.5	100	99.7
10	94.3	98.2	95.8
15	93.8	97.5	99.6

### Validation of the Process for Clinical use

Mean decay-corrected radiolabeling yields after optimization were 96.0 ± 1.7% (see Supplementary Table 1). The mean percentage of decay-corrected radioactivity remaining on the filter at the end of the purification step was 1.2 ± 1.2% (see Supplementary Table 1).

The results of all the controls performed on the two series of three [^68^Ga]Ga-MAA suspensions are shown in the Table 2. Mean [^68^Ga]Ga-MAA radiochemical purity was 99.0 ± 0.6%. Mean percentage of particles smaller than 3 μm was 1.3 ± 0.8% at the end of the synthesis process. Germanium-68 percentage of each obtained [^68^Ga]Ga-MAA suspension was compliant with the level required in EU Pharmacopeia monograph (i.e., inferior to 0.001% of total radioactivity). The pH of all tested solutions was 4.4. [^68^Ga]Ga-MAA suspensions were sterile and the endotoxin content was below 5 IU/mL (EU Pharmacopeia specifications: <12.5 IU/mL).

Three hours after the end of the synthesis, mean [^68^Ga]Ga-MAA radiochemical purity was 98.9 ± 1.1%, and mean percentage of particles with a size inferior to 3 μm was 1.3 ± 0.8%.

### MAA Integrity at the End of the Labeling

The size of MAA from the commercial kit before labeling and the size of [^69^Ga]Ga-MAA produced with the process for clinical use ranged from 15 to 75 μm (see Supplementary Figure 1). In all samples tested, 70% or more MAA size was between 25 and 50 μm. No particles size was superior to 100 μm, in line with EU Pharmacopeia sheets for [^99m^Tc]Tc-MAA.

**Figure 4:**
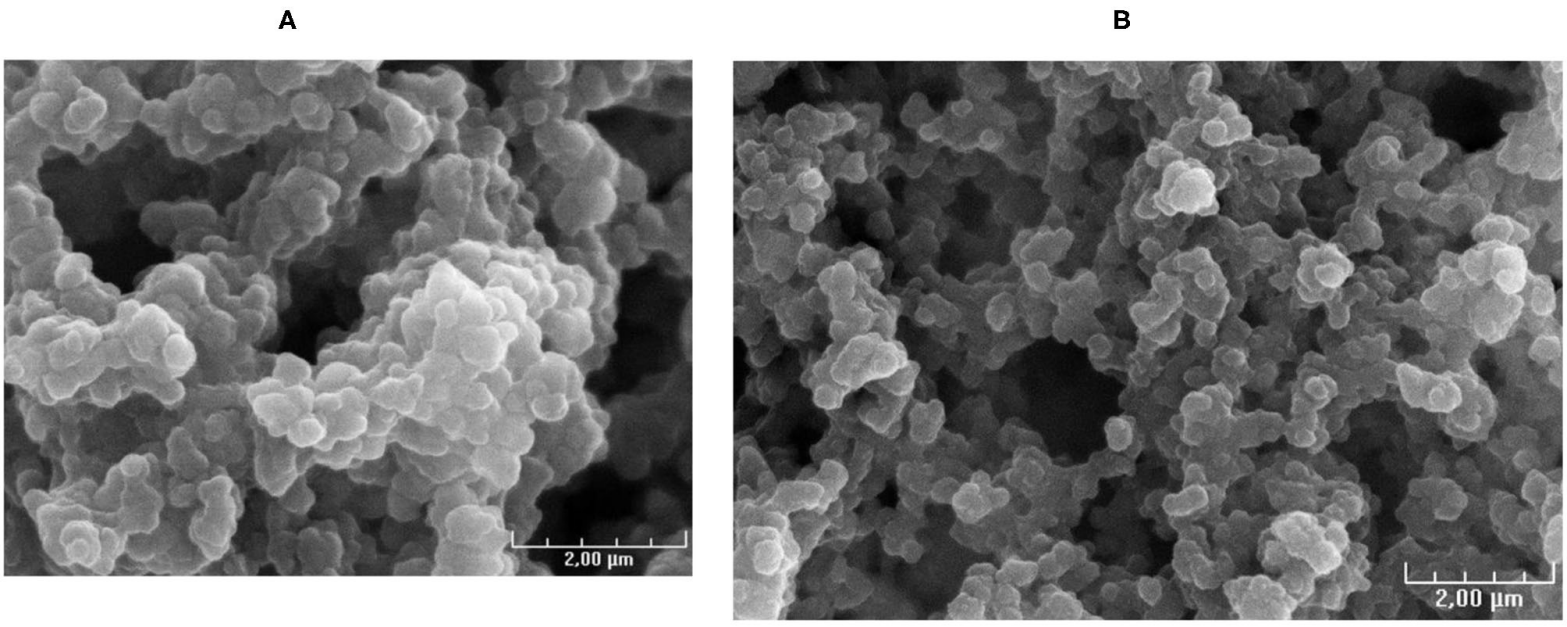
SEM images of: **(A)** MAA before labeling and **(B)** stable gallium MAA from synthesis with the process for clinical use.

Visual analysis of SEM images of MAA before labeling and [^69^Ga]Ga-MAA from synthesis with a process for clinical use showed similar MAA morphology (Figure 4).

### Labeled MAA Suspension Tin Rate

Mean tin total amount in the final product was 0.0232 ± 0.005 mg, as compared with 0.21 mg in the MAA commercial kit before labeling (see Supplementary Table 2).

## Discussion

We developed a fully automated process to produce GMP [^68^Ga]Ga-MAA for clinical use, using a non-modified MAA commercial kit and a direct gallium-68 eluate. The process includes an innovative automated process for [^68^Ga]Ga-MAA purification, based on the use of a 0.22-μm pore size filter with a passage of the suspension with specific settings. The full process is simple, fast (15 min), and allows isolating the gallium-68 eluate required for the carbon nanoparticles production. The process demonstrated high reliability and reproducibility, with high radiolabeling yield (>95%).

We tested various synthesis parameters based on previously published data, that reported reaction mixture heating temperatures ranging from 50 to 115°C (16, 19), heating times up to 20 min (5, 17), and pH ranging from 4 to 6.5 (5, 17, 19). In this work, out of the four pH tested, three (3.8, 4.3, and 4.8) were able to maintain the organoleptic properties of the MAA between 50 and 70°C. At pH from 4.3 to 5.3, a heating temperature of 80°C or more altered the MAA quality. These data are in line with a study from Amor-Coaraza et al., which showed that a heating stage of 15 min at a temperature superior to 75°C resulted in a higher concentration of small particles in the reaction mixture, which could be explained by the rupture of large macroaggregates (15, 19). Accordingly, to limit the influence of parameters variation on the labeling yields, we retained intermediate values, i.e., a reaction mixture pH of 4.3 and a heating temperature of 60°C. In order to decrease the synthesis time, three radiolabeling times (= heating times) were tested: 5, 10, and 15 min. Similar mean decay corrected radiochemical yields (93.5% for 5 min, 94.3% for 10 min, and 93.8% for 15 min) and [^68^Ga]Ga-MAA suspensions radiochemical purity (100% for 5 min, 98.2% for 10 min, and 97.5% for 15 min) were found with the three heating stages tested. Accordingly, a 5-min heating stage was maintained for the synthesis process for clinical use.

Our process uses non-washed MAA from a commercially available kit. This is a difference with many groups (5, 12, 17–19) that carried out MAA labeling with washed MAA in order to remove the excess of free albumin to improve the labeling yield. On the other hand, Mueller et al. detected no difference in the labeling efficiency using non-washed MAA as compared with pre-washed MAA (16). In order to simplify the synthesis process, we made the choice to use unmodified MAA kits. This resulted in high decay-corrected radiochemical yields (96.0 ± 1.7%), which are superior to the radiochemical yields reported with washed MAA, ranging from 78 to 90% (5, 12, 17–19).

Still with the aim to simplify the process, a direct gallium-68 eluate was selected. A pre-purification of the gallium-68 eluate was proposed by several groups to remove the metallic impurities and germanium-68 and improve the labeling yields (5, 12, 21). Amor-Coarasa et al. (19) compared both processes, and reported that the introduction of a gallium-68 pre-purification system improved the labeling yield significantly as compared with a direct use of a gallium-68 eluate (78.3 ± 3.1% as compared with 84.1 ± 3.4%). However, without eluate pre-purification, our mean labeling yield was 96.0 ± 1.7%, in line with previous published data (17). Furthermore, the direct use of gallium-68 eluate could have increased the germanium-68 level in the final product. However, our purification process demonstrated very low germanium-68 levels in our [^68^Ga]Ga-MAA suspensions (<0.001% of total radioactivity).

The key step of the synthesis, and the innovative point of this work, is the purification process. Maus et al. proposed a manual purification stage, using a Sep Pack C18 cartridge which was washed two times with sterile water to remove [^68^Ga]Ga-MAA (17). Some teams used a centrifuge to purify the obtained suspension of [^68^Ga]Ga-MAA (5, 12, 18–20). The purification stage must eliminate the free gallium-68 from the final product. This step is critical because the filter or the column has to be able to trap the [^68^Ga]Ga-MAA, and in turn, to release the product with a good yield. In this work, we aimed at: (a) passing the synthesized [^68^Ga]Ga-MAA particles on a syringe filter membrane which membrane composition, diameter, and pore size were chosen so that the [^68^Ga]Ga-MAA particles are trapped, while impurities from the bulk solution are not retained, said impurities essentially consisting of free metallic impurities, free gallium-68, and stannous chloride present in the MAA commercial kit; (b) untrapping the [^68^Ga]Ga-MAA particles from the syringe filter using a saline solution passing through the syringe filter in the opposite direction of the trapping movement and providing the final injectable bulk solution into a vial. The gravity is a key factor to improve the release of [68Ga]Ga-MAA from the filter. That is why the suspension was passed from the bottom to the top and vice versa for elution. Better untrapping results were obtained with a vented filter. The reason may be that the use of a vented filter limited the pressure when the [^68^Ga]Ga-MAA solution passed through the filter. Thus, [^68^Ga]Ga-MAA was only deposed on the filter and not trapped in the filter. With this process, only a low radioactivity percentage remains on the filter (0.3–3.5%) leading to a high synthesis yield (93–98%). Furthermore, the final product demonstrated high quality with mean radiochemical purity of the [^68^Ga]Ga-MAA suspensions of 99.0 ± 0.6%. Microscopic examination showed the ^68^Ga-labeled MAA particles remain within their original size and morphology. The purification stage was also able to decrease by 10 times the tin level in the final product in comparison with the MAA commercial kit. Finally, another advantage of this technique is that it is a fully automated process which reduces the operator's radiation exposure.

A limit of this work is that our process was developed for the use of an Eckert and Ziegler ^68^Ge/^68^Ga generator. The use of another generator, such as an IRE ^68^Ge/^68^Ga generator, should be possible, but as the eluate volume may be smaller (1.1 mL with the IRE generator as compared with 5 ml with the Eckert and Ziegler generator), it will not be possible to take out the aliquot for the labeling of carbon nanoparticles and the molarity of the sodium acetate solution should be appropriate to respect the reaction media pH.

What are the clinical implications of our findings? This process offers several advantages to ease the implementation of lung PET/CT imaging in nuclear medicine. The labeling procedure is very simple, GMP compliant, and fully automated. The cassette is directly mounted onto the final product vial without a sterile filter for final sterilization. The process is reliable and reproducible with very good mean decay-corrected radiochemical yields (96.0 ± 1.7%). According to the age of the ^68^Ge/^68^Ga generator, providing a gallium-68 eluate activity ranging from 720 to 360 MBq for 3 mL, the total activity of the final product ranges from 650 to 300 MBq/10 mL, which is largely sufficient to perform multiple perfusion PET/CT scans with only one preparation. Indeed, 50 MBq of [^68^Ga]Ga-MAA are usually administrated for a V/Q PET/CT scan, and much lower for a perfusion PET/CT scan without ventilation imaging. Furthermore, [^68^Ga]Ga-MAA suspension was stable *in vitro* at least 3 h after the end of the synthesis.

## Conclusion

We developed a fully automated process to produce GMP [^68^Ga]Ga-MAA for clinical use, using a non-modified MAA commercial kit and a direct gallium-68 eluate, and including an innovative automated process for [^68^Ga]Ga-MAA purification. The full process is simple, fast (15 min), reliable, and reproducible, and allows isolating the gallium-68 eluate required for the ^68^Ga-labeled carbon nanoparticles production.

This automated process may facilitate the implementation of lung PET/CT imaging in nuclear medicine departments and increase its accessibility to patients with lung disease.

## Data Availability Statement

The original contributions presented in the study are included in the article/Supplementary Material, further inquiries can be directed to the corresponding authors.

## Author Contributions

All authors contributed to the study conception and design. [^68^Ga]Ga-MAA syntheses and quality controls were performed by FB-B, SH, VG, PE, and JM. The first draft of the manuscript was written by FB-B and P-YL and all authors commented on previous versions of the manuscript. All authors read and approved the final manuscript.

## Conflict of Interest

The authors declare that the research was conducted in the absence of any commercial or financial relationships that could be construed as a potential conflict of interest.

## Publisher's Note

All claims expressed in this article are solely those of the authors and do not necessarily represent those of their affiliated organizations, or those of the publisher, the editors and the reviewers. Any product that may be evaluated in this article, or claim that may be made by its manufacturer, is not guaranteed or endorsed by the publisher.
