# Supplementary material for: Fully Automated 68Ga-Labeling and Purification of Macroaggregated Albumin Particles for Lung Perfusion PET Imaging
Source: Front Nucl Med. 2021 Nov 18;1:778191. doi: 10.3389/fnume.2021.778191 (PMC11440869; doi:10.3389/fnume.2021.778191)
Supplement: Supplementary file 1 [file Data_Sheet_1.docx]

**Supplementary Figure 1** percentage of MAA (dark grey) and [^69^Ga]Ga –MAA (light grey) according to the size in µm.
