# Supplementary material for: Fully Automated 68Ga-Labeling and Purification of Macroaggregated Albumin Particles for Lung Perfusion PET Imaging
Source: Front Nucl Med. 2021 Nov 18;1:778191. doi: 10.3389/fnume.2021.778191 (PMC11440869; doi:10.3389/fnume.2021.778191)
Supplement: Supplementary file 2 [file Table_1.docx]

**Supplementary table 1** Decay corrected radiolabelling yield of three syntheses performed without purification stage and six syntheses performed with the automated process for clinical use. Radioactivity staying on the filter at the end of the synthesis for the six procedures performed with the process for clinical use.

|  | Automated process without purification stage | Automated process for clinical use | |
| --- | --- | --- | --- |
|  | Radiolabelling yield (%) | Radiolabelling yield (%) | Radioactivity on the filter (%) |
|  | 88 | 96 | 3.5 |
|  | 85 | 98 | 1.2 |
|  | 89 | 97 | 1.4 |
|  |  | 96 | 0.3 |
|  |  | 93 | 0.4 |
|  |  | 96 | 0.3 |
| Mean | 87.3 | 96 | 1.2 |
| Standard deviation | 2.1 | 1.7 | 1.2 |
