# Supplementary material for: Fully Automated 68Ga-Labeling and Purification of Macroaggregated Albumin Particles for Lung Perfusion PET Imaging
Source: Front Nucl Med. 2021 Nov 18;1:778191. doi: 10.3389/fnume.2021.778191 (PMC11440869; doi:10.3389/fnume.2021.778191)
Supplement: Supplementary file 3 [file Table_2.docx]

**Supplementary table 2** Results of tin dosage in four [^68^Ga]Ga-MAA suspensions produced with the process for clinical use. The dosages were performed using High Resolution Inductively Coupled plasma Mass Spectrometry.

| Sample | Tin concentration in the final product(mg/g) | Total amount of tin in the final product (mg) | Tin total amount in the MAA commercial kit before labelling (mg) |
| --- | --- | --- | --- |
| 1 | 2.96 | 0.0296 | 0.21 |
| 2 | 2.44 | 0.0244 | 0.21 |
| 3 | 1.80 | 0.0180 | 0.21 |
| 4 | 2.07 | 0.0207 | 0.21 |
| Mean | 2.3 | 0.0232 |  |
| Standard deviation | 0.5 | 0.0050 |  |
